# Connecting telomere maintenance and regulation to the developmental origin and differentiation states of neuroblastoma tumor cells

**DOI:** 10.1186/s13045-022-01337-w

**Published:** 2022-08-27

**Authors:** Eun Young Yu, Nai-Kong V. Cheung, Neal F. Lue

**Affiliations:** 1 Department of Microbiology & Immunology, W. R. Hearst Microbiology Research Center, Weill Cornell Medicine, 1300 York Avenue, New York, NY 10065 USA; 2 Department of Pediatrics, Memorial Sloan Kettering Cancer Center, 1275 York Avenue, New York, NY 10065 USA; 3 Sandra and Edward Meyer Cancer Center, Weill Cornell Medicine, 1300 York Avenue, New York, NY 10065 USA

**Keywords:** Neuroblastoma, Telomeres, Telomere maintenance mechanisms, Telomerase, ALT, Tumor cell differentiation, ADRN, MES, Immunogenicity, Telomere trimming, Telomere DNA damage

## Abstract

A cardinal feature that distinguishes clinically high-risk neuroblastoma from low-risk tumors is telomere maintenance. Specifically, neuroblastoma tumors with either active telomerase or alternative lengthening of telomeres exhibit aggressive growth characteristics that lead to poor outcomes, whereas tumors without telomere maintenance can be managed with observation or minimal treatment. Even though the need for cancer cells to maintain telomere DNA—in order to sustain cell proliferation—is well established, recent studies suggest that the neural crest origin of neuroblastoma may enforce unique relationships between telomeres and tumor malignancy. Specifically in neuroblastoma, telomere structure and telomerase activity are correlated with the adrenergic/mesenchymal differentiation states, and manipulating telomerase activity can trigger tumor cell differentiation. Both findings may reflect features of normal neural crest development. This review summarizes recent advances in the characterization of telomere structure and telomere maintenance mechanisms in neuroblastoma and discusses the findings in the context of relevant literature on telomeres during embryonic and neural development. Understanding the canonical and non-canonical roles of telomere maintenance in neuroblastoma could reveal vulnerabilities for telomere-directed therapies with potential applications to other pediatric malignancies.

## Introduction: emerging roles of tumor cell differentiation and telomeres in neuroblastoma malignancy

Neuroblastoma or NB, derived from the embryonal neural crest cells, is the most common solid tumor in children and represents ~ 6% of all pediatric tumor diagnosis [1, 2]. Despite improvement in therapies over the years, it remains one of the most aggressive and lethal pediatric tumors, accounting for 10% of mortality and ~ 15% of all pediatric cancer deaths. NB is known to have remarkably heterogeneous clinical outcomes ranging from spontaneous remission to lethal progression [1, 2]. Accordingly, accurate assessment of prognosis and stratification of patients into different risk groups are crucial for cure while reducing unnecessary toxicities [3, 4]. Indeed, low-risk NB patients have excellent outcomes with minimal or no treatments. By contrast, high-risk patients have an overall cure rate of about 50% despite undergoing multi-modal therapies including surgery, chemotherapy, radiotherapy, immunotherapy, and targeted therapy [5, 6].

### NB tumor cell differentiation states and malignancy

**Fig. 1:**
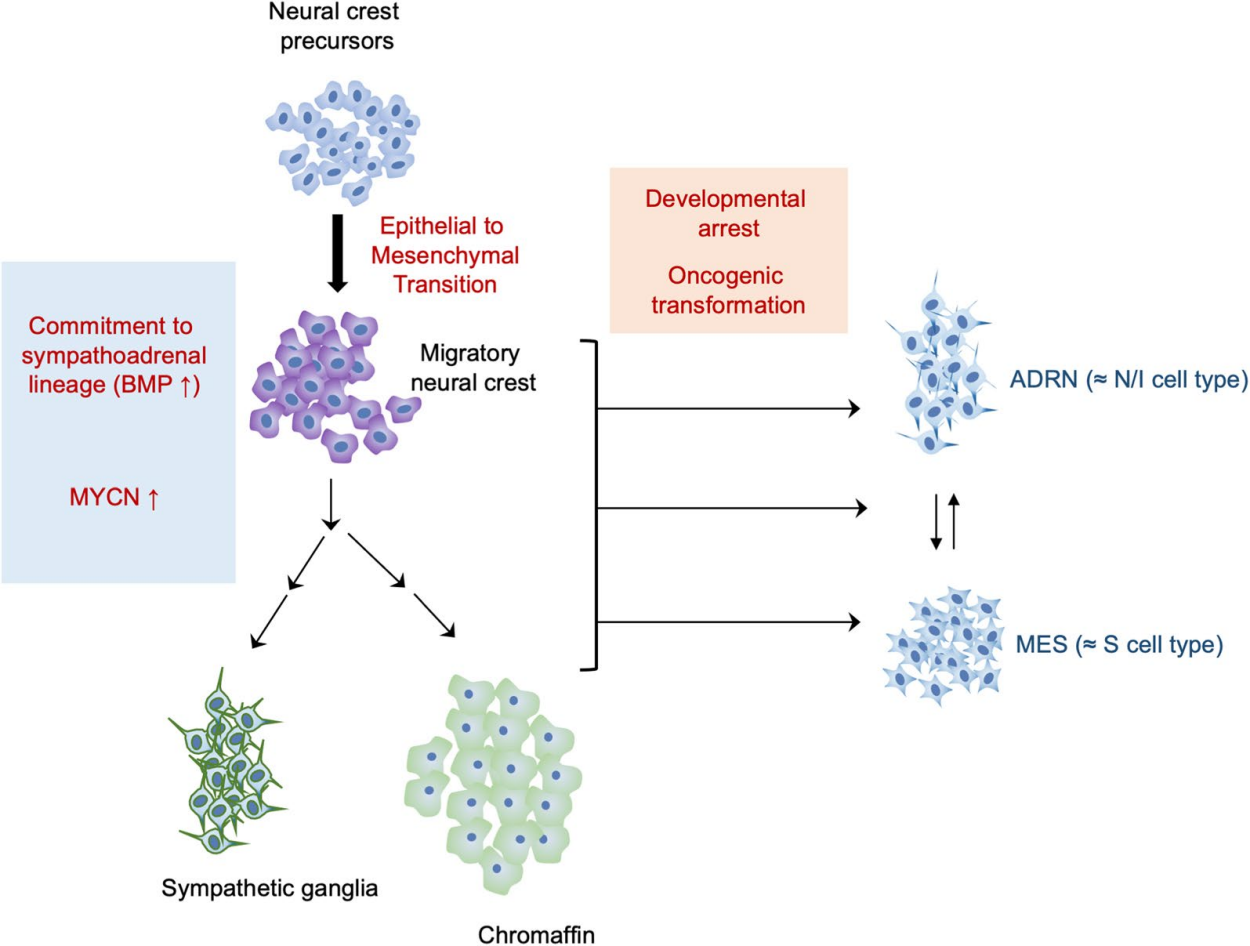
The developmental origin of neuroblastoma and its relationship to the heterogeneity of tumor cell differentiation. A schematic illustration of the developmental pathway of the neural crest cells that give rise to neuroblastoma is presented [1, 2]. The tumor-initiating cells are thought to have committed to the sympathoadrenal lineages and to be on the pathways toward differentiating into various mature cell types (e.g., sympathetic ganglia and chromaffin). The ADRN and MES cell states in NB tumors (which evidently correspond to previously defined N/I and S cell types, respectively) may reflect different stages of neural crest differentiation along these pathways

NB is a developmental malignancy that originates from the sympathoadrenal neural crest, notable for its plasticity in responding to environmental cues [7, 8] (Fig. 1). This developmental origin is a likely blueprint for its clinical heterogeneity. The pathway of neural crest differentiation and maturation is a complicated one involving the sequential activities of multiple transcription factors. Disruption of different steps along the pathway may lead to tumors with distinct phenotypes and malignant potentials. Perhaps not surprisingly, NB tumor cells manifest phenotypic diversity reminiscent of the plasticity of neural crest progenitor cells. Early studies of isogenic cell lines derived from the same tumor segregated tumor cells into three cell types based on morphologies and biochemical markers: N (neuroblastic)-type cells have scant cytoplasm and neuritic processes; S (substrate-adherent)-type cells have extensive cytoplasm and resemble non-neuronal precursor cells; and I (intermediate)-type cells are uncommitted to either N or S cells [9–12]. These cell types are interconvertible using pharmacologic agents, and they display varying degrees of tumorigenicity in animal models. In particular, the I cells (malignant NB stem cells) were found to be more malignant than N or S cells [10]. More recently, through expression and epigenetic profiling, NB tumor cells were reclassified into two major groups, named the ADRN (adrenergic) and MES (mesenchymal) cells (Fig. 1). These two cell types can be distinguished by the activation of lineage-specific super-enhancers, which control the expression of ADRN- and MES-signature genes [13–15]. For examples, ADRN-specific super-enhancers are associated with the expression of adrenergic differentiation makers such as *PHOX2A*, *PHOX2B,* and *DBH* [14], whereas MES-specific super-enhancers are similar to those found in neural crest-derived cells [14, 16]. Consistent with phenotypic plasticity, MES and ADRN cells can trans-differentiate from one cell type into the other [14, 17]. Comparison of the earlier morphologic/biochemical and the later genetic classifications revealed broad congruence between cell types from both schemes, with the N/I-type cells resembling the ADRN cells and the S-type cells resembling MES cells [9, 14, 17] (Fig. 1). For example, both S and MES cells harbor high levels of cytoskeletal proteins associated with the mesenchymal phenotype. Of particular interest, the differentiation state of tumor cells has been implicated in disease progression and treatment response and may play a role in determining the risk profile. While the ADRN cells are more chemo-sensitive and appear to be predominant at diagnosis, the MES cells are more chemo-resistant and may be enriched in relapse and in metastatic diseases [14, 15, 18, 19]. The relatively “quiescent” behavior of MES cells is reminiscent of dormant clones that became explosive at recurrence. The ability of the ADRN and MES cell types in vitro to “transdifferentiate” highlights the plasticity of the cell states, which are not irreversibly fixed by lineage assignments, but interconvertible depending on environmental cues or pressures.

### Genetic alterations in NB tumors

**Fig. 2:**
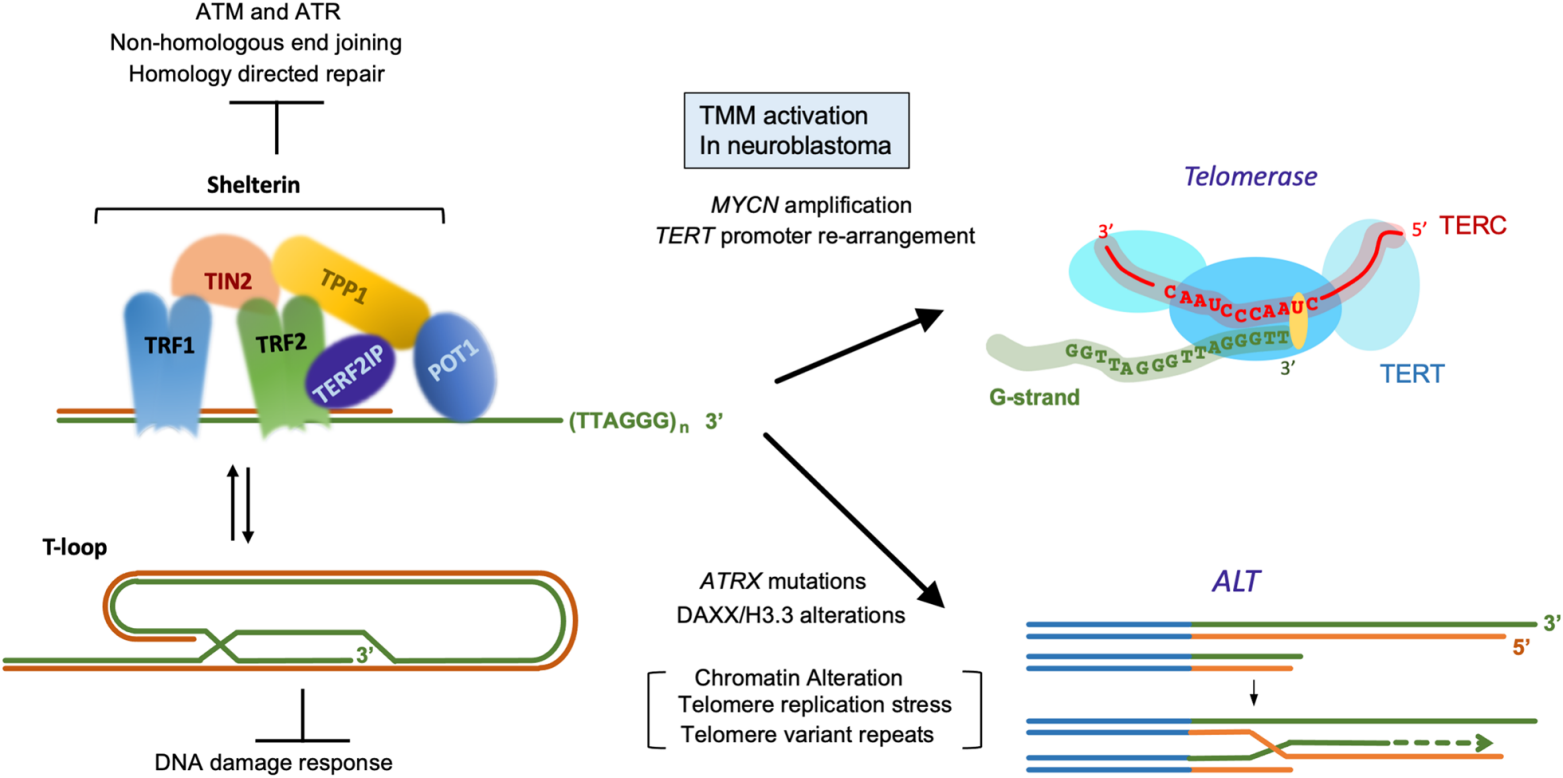
Telomere structure and maintenance mechanisms. The basic structure of telomere DNA (a double-stranded telomere repeat region that terminates in a 3′ single-stranded overhang) and the major telomere protection complex (shelterin) are illustrated on the left and the two telomere maintenance mechanisms are shown on the right [46, 47, 63]. Shelterin comprises a network of six proteins that collectively bind to both the double-stranded telomere repeats and the 3’-overhangs. This special nucleoprotein complex stabilizes chromosome ends by inhibiting DNA damage response and DNA repair pathways. Telomere DNA can also adopt the “T-loop” conformation in which the 3’-overhang forms base pairs with a more proximal region of telomere repeats. This T-loop structure also suppresses the DNA damage response. Telomerase is a special reverse transcriptase comprised of a catalytic protein component (TERT) and a template RNA (TERC). ALT is a recombination-based telomere elongation pathway that resembles break-induced replication. In high-risk neuroblastoma, recurrent genomic aberrations are tightly linked to either the up-regulation of telomerase or activation of ALT. The telomerase pathway is primarily up-regulated by *MYCN* amplification or *TERT* promoter re-arrangement, whereas ALT is often activated by alterations in ATRX/DAXX/H3.3 and is associated with telomere replication stress, chromatin de-condensation, and elevated levels of telomere variant repeats

The clinical heterogeneity of NB stems also from the distinct underlying genetic alterations (as reviewed in [20]). However, while the genomic landscape of NB is complex, some mutations and structural alterations are recurrently associated with high-risk NB (HR-NB). The driver mutations that had been identified include those in *MYCN, ATRX (α thalassemia/mental retardation syndrome X-linked), TERT,* and *MDM2-CDK4* [21–24]. These can be coupled with collaborative mutations in RAS-MAPK, PI3K-mTOR and TP53 pathways [21, 25, 26]. In a recent clonal analysis of tumor samples, the amplification of *MYCN* occurs in ~ 20 to 30%, *ATRX* mutations or deletions in ~ 10%, *TERT* mutations in ~ 10%, and *MDM2-CDK* mutations in ~ 2% of HR-NB [27]. Still, nearly 50% of HR-NB do not carry these driver mutations, but only segmental or numerical copy number variations. Chromosomal aberrations (deletion of 1p and 11q and gain of 17q) are common in HR-NB and implicated in unfavorable outcomes [28–31]. Mutations of *ALK* (anaplastic lymphoma kinase) are found scattered among these driver mutational types and in most cases of familial NB [32, 33]. Although the mechanisms are not fully understood, these genetic aberrations are thought to alter the developmental and differentiation pathways of normal neural crest, thereby triggering malignancy. MYCN, for example, is transiently expressed in migrating neural crest cells [34], and overexpression of MYCN by gene amplification not only enhances proliferation but also compromises differentiation [35]. Inactivating mutations in *ATRX* represent another genetic alteration associated with a distinct clinical cohort; these cases present predominantly in older children and young adults and manifest a chronic and progressive clinical course with high overall mortality [36]. ATRX is a multi-functional chromatin remodeling factor implicated in both transcription and replication, as well as in suppressing the ALT (alternative lengthening of telomeres) pathway [37] (Fig. 2). Mutations in *ATRX*, like *MYCN* amplification, could conceivably alter the differentiation of neural crest, thereby promoting oncogenic transformation.

### Telomere maintenance in NB malignancy

Notably, despite the heterogeneity of the mutational landscape, recent studies suggest a common mechanism by which multiple genetic alterations promote HR-NB, namely through telomere maintenance. Specifically, analysis of extensive tumor collections confirmed the prevalence of three key mutational driver types in HR-NB, namely *MYCN* amplification, *ATRX* inactivation, and rearrangement of the *TERT* promoter [22, 38, 39]. All three genetic alterations are strongly associated with TMM (telomere maintenance mechanism) activation, supporting a shared mechanism for driving malignancy (Fig. 2). Indeed, profiling of tumor collections revealed a strong linkage between the presence of TMM (high telomerase or ALT activity) and poor prognosis for HR-NB [21, 40].

Notably, studies to date have highlighted tumor cell differentiation and telomere maintenance as two separate determinants of NB disease biology. However, a recent report suggests that these determinants are not independent, but instead mechanistically connected [17]. In particular, the ADRN and MES cell types were found to exhibit dramatically different levels of telomere-related factors, and inhibition of telomerase triggered the conversion of ADRN into MES cell types in a reversible manner [17]. While unexpected, a potential role of telomeres and telomerase in NB differentiation is plausible in light of multiple studies linking telomere proteins to neural development and neural differentiation. For example, one of the essential telomere proteins, TRF2 (telomeric repeat-binding factor 2), was shown to regulate neural cell differentiation and neural protection partly via non-telomeric pathways [41–43]. In addition, telomerase activity and the catalytic protein subunit (TERT) have been reported to function in neural development and developing brain neurons [44, 45]. Thus, telomere proteins and telomerase may have the potential to alter the differentiation state of neural crest-derived NB tumor cells in accordance with the developmental origin of these cells. In the ensuing sections, we outline current understanding of the roles of telomeres and TMMs in cancers and describe in greater detail how they impact on NB disease biology. We summarize studies that support a mechanistic connection between telomeres and NB differentiation, and we examine this connection in relation to other studies that link telomeres and telomerase to normal neural development and differentiation. Finally, we discuss the implications of this connection in the development of new biomarkers and therapies for HR-NB.

## Telomere structure and telomere maintenance in normal and cancer cells

Telomeres, the nucleoprotein structures at eukaryotic chromosome ends, consist of numerous copies of a simple short DNA repeat as well as a collection of telomere-associated proteins [46] (Fig. 2). Maintenance of telomere length and structure is crucial for cell proliferation and genomic integrity in both somatic and germ cells (as reviewed in [46, 47]). The repeat sequence of mammalian telomeres is TTAGGG/CCCTAA, which exists mainly as double-stranded DNA repeats (4 ~ 12 kb base pairs), but which terminates in a single-stranded 3′ overhang on the G-strand (50 ~ 400 bp nucleotides). This 3′ overhangs can invade the proximal double-stranded telomeric DNA to form T-loops, which prevent the activation of DDR (DNA damage response) and allow cells to distinguish normal chromosome ends from DSBs (double-strand breaks) [48, 49] (Fig. 2). The main telomere-associated protein complex is shelterin, a six-protein assembly (TRF1-TRF2-POT1-TIN2-TERF2IP-TPP1). Among the shelterin components are two proteins that directly bind double-stranded telomere repeats, TRF1 (telomeric repeat-binding factor 1) and TRF2. TRF1 plays a major role in maintaining telomeric DNA, whereas TRF2 is important for telomere protection, in part by promoting T-loop formation. In addition, TRF2 recruits TERF2IP (TRF2-interacting protein 1, also known as RAP1) [50], another telomere-protective factor, through protein–protein interactions. The major 3’ overhang-binding protein is POT1 (protection of telomeres protein 1), which cooperates with TIN2 (TRF2- and TRF1-interacting nuclear protein 2) and TPP1 (TIN2 interacting protein 1) to bridge single-stranded and double-stranded telomere DNAs through a network of interactions (i.e., POT1-TPP1-TIN2-TRF1 and POT1-TPP1-TIN2-TRF2). Formation of this functional, six-protein shelterin complex at telomeres protects telomeres and promotes genome stability by suppressing ATM (Ataxia telangiectasia mutated)- and ATR (ATM and Rad3-related)-dependent pathways, homologous recombination, and c-NHEJ (classical non-homologous end joining) at chromosome ends (Fig. 2).

Eukaryotic linear chromosomes experience loss of telomere DNA during each round of cell division due to incomplete end replication on the lagging-strand [51]. In addition, because the G-rich telomere repeats can form a variety of secondary structures that hamper replication fork progression, telomeres can experience stochastic truncations [51, 52]. Another pathway that can induce rapid telomere loss is “telomere trimming,” a recombination-based telomere shortening pathway [53, 54]. This pathway appears to be especially active in stem cells and germ cells, and is thought to be a homeostatic mechanism that prevents excessive telomere elongation [53, 54]. Finally, telomere DNA damage, including oxidative damage, has been shown to result directly in accelerated telomere loss [55, 56]. Because an adequate amount of telomere DNA is required to suppress DNA damage response and sustain cell proliferation, the combined effect of telomere shortening pathways may eventually compromise the capacity of cells to divide.

To overcome the growth suppressing effect of telomere shortening, the majority of cancer cells activate one of two TMMs to elongate telomeric DNA: telomerase and ALT (Fig. 2). The predominant TMM employed by human cancers is telomerase, a ribonucleoprotein complex that extends the G-strand by copying an RNA template component (TERC) using a catalytic reverse transcriptase (TERT). Telomerase activity is repressed in most normal somatic cells but up-regulated in the 85 to 90% of human cancers [57]. The up-regulation of telomerase is due primarily to elevated *TERT* transcription, which can be accomplished by a variety of mechanisms, including point mutations in the *TERT* promoter [58, 59], *TERT* gene rearrangement [22, 39], *TERT* gene amplification [60, 61], overexpression of MYC [62], or *MYC* gene amplification [39].

The second TMM, detected in 10 ~ 15% of human tumors, is a recombination-dependent mechanism named ALT [63, 64]. ALT resembles BIR (break-induced DNA replication) and is mechanistically complex; it encompasses both a RAD52-dependent and a RAD52-independent pathway [65, 66]. The activation mechanisms for ALT are still unclear but several predisposing factors have been identified. It has been shown that genetic mutations in *ATRX*, *DAXX* (*death domain-associated protein*), and histone variant *H3.3* are involved in ALT activation in many different malignancies [67–70]. In an early study, the great majority of ALT cell lines were shown to be *ATRX*-defective [71]. However, more recent, large-scale analyses of cell lines and tumor samples identified a substantial fraction of ALT-positive samples that do not harbor *ATRX* mutations [72–74]. Both ATRX and DAXX are implicated in chromatin remodeling and have been suggested to suppress ALT activation by regulating telomeric chromatin. Telomere chromatin changes are thought to increase accessibility and telomere replication stress, which if persistent, will ultimately trigger aberrant recombination [75] (Fig. 2). One characteristic marker of ALT cells is APB (ALT-associated promyelocytic leukemia (PML) body), which contains telomeric DNA, shelterin complex, DNA replication- and recombination-related proteins. The weight of evidence points to APBs as the active sites for telomere DNA synthesis by recombination [66]. Another characteristic of ALT is high levels of single- and double-stranded ECTRs (extrachromosomal telomeric repeats). One of the ECTR species, telomeric C-circle (circular DNA that consists of uninterrupted C-strand and nicks/gaps on the G-strand), is frequently used as a quantitative marker of ALT activity [76]. ALT telomeres also contain high levels of telomere variant repeats, which recruit nuclear receptors to facilitate telomere recombination and exacerbate genomic instability [73, 77, 78].

## Telomeres and TMMs in neuroblastoma

As noted before, NB is remarkable for its heterogeneous clinical outcomes ranging from spontaneous regression to uncontrollable progression [1, 2]. Multiple studies over the past two decades have uncovered roles of telomere-specific features in the risk factors and clinical outcomes for NB. For example, a longer telomere length is associated with worse prognosis, and heterogeneous telomere length within individual NB is strongly related to progression and death [79, 80]. In addition, common genetic variants associated with longer leukocyte telomere lengths have been shown to confer risk for NB and other childhood cancers [81]. More importantly, recent studies of NB tumor samples highlight TMMs as a key prognostic indicator. Patients with HR-NB often experience relapse and the majority of relapsed HR-NB manifests activation of TMMs, i.e., either telomerase or ALT [21, 40]. While the frequencies vary between study cohorts, in one recently analysis, ~ 50% and ~ 25% of HR-NB show evidence of high telomerase and ALT activities, respectively [40]. TMMs could add to known genetic prognosticators; for example, mutations in the RAS and p53 pathways are more unfavorable with TMM activation in HR-NB, but lose their effect in TMM-negative low-risk NB [21].

**Fig. 3:**
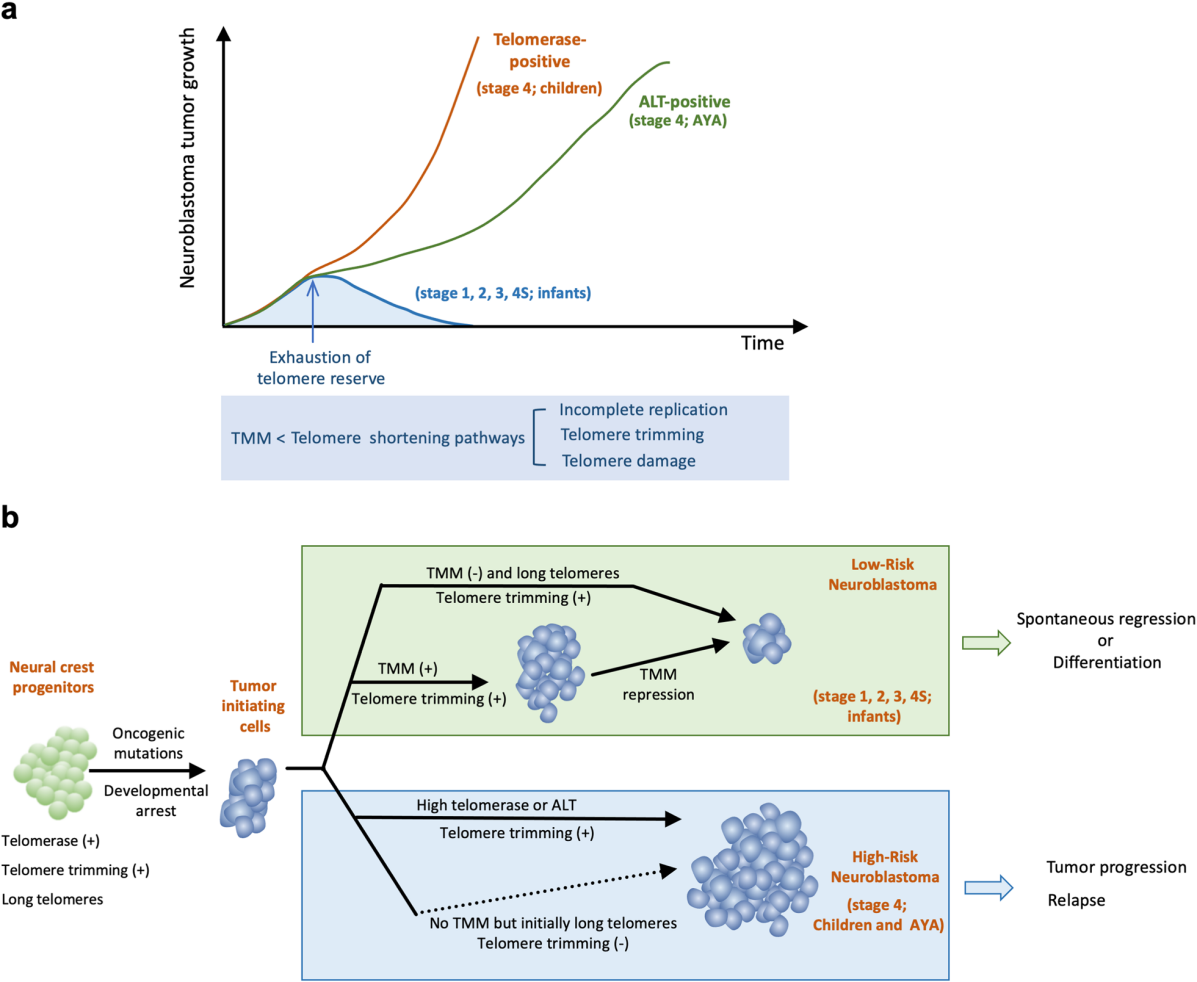
Telomere maintenance in the development of high-risk and low-risk neuroblastoma. **a.** A model for the growth of NB tumors that belong to different risk groups. Low-risk NB (stage 1, 2, 3 and 4 s) has no TMM; hence, the progressive erosion of telomeres (due to incomplete end replication, telomere trimming, and telomere DNA damage) eventually results in the exhaustion of telomere reserve and loss of proliferative capacity. HR-NB tumors (stage 4) harbor either telomerase or ALT and are able to proliferate indefinitely by counteracting telomere loss. Telomerase-positive NB tumors are faster growing and present predominantly in children, whereas ALT-positive tumors are slower growing and present predominantly in adolescents and young adults. **b.** A more detailed model on how dynamic changes in telomere trimming activity and telomere maintenance mechanisms during and after oncogenic transformation may influence the growth of NB tumors. The neural crest progenitor cells are proposed to harbor both telomere trimming and telomerase activity. In low-risk NB (green-shaded box), the tumor-initiating cells may either harbor no TMM or turn off telomerase expression in accordance with the developmental program in normal neural crest. The inability to counteract telomere shortening mechanisms leads eventually to short telomeres that are unable to support tumor growth. In contrast, HR-NB can sustain proliferation by either activating TMMs or (in a minority of cases) by repressing telomere trimming completely prior to shutting off telomerase. Low-risk NB spontaneously regress or differentiate when the telomere reserve is insufficient to sustain cell proliferation, whereas high-risk NB tends to progress and cause relapse

Consistent with the crucial importance of TMMs in NB, three of the most frequent genetic alterations in HR-NB appear to represent mutually exclusive drivers of the disease, and each alteration is mechanistically linked to TMM activation. Two of these genetic alterations, i.e., *TERT* mutations (*TERT* promoter rearrangement and *TERT* amplification) and *MYCN* amplification, up-regulate telomerase activity [38, 39]. The promoter re-arrangement presumably links the *TERT* gene to an ectopic enhancer, whereas MYCN binds directly to the *TERT* promoter to stimulate transcription. Notably, while *TERT* mRNA is typically over-expressed in *MYCN*-amplified NB, there are exceptions [40], suggesting that additional genetic or epigenetic factors are involved in facilitating MYCN-mediate TERT expression. The third alteration, *ATRX* mutations, predisposes cancer cells to ALT activation and is present in 55 to 60% of ALT-positive NB [75]. Interestingly, even though a significant fraction of ALT-positive NB tumors does not harbor *ATRX* mutations, such tumors often exhibit low ATRX protein expression, suggesting a recurrent pathway for ALT activation that involves ATRX loss of function [40, 82]. Notably, each TMM is associated with a distinct clinical presentation and disease course (Fig. 3a). Telomerase-positive NB tumors are primarily diagnosed in children > 18 months of age and are characterized by a rapid and aggressive clinical course. In contrast, ALT-positive NB tumors predominantly affect adolescents and young adult (AYA) patients and are characterized by a chronic but steadily progressive clinical course with ultimate mortality [36]. Thus, beyond conferring tumor cells with unlimited proliferation potential, TMMs may also modulate the growth rate of tumors [83]. In contrast to high-risk NB, low-risk NB cases are most often found in infants and are characterized by locoregional or stage 4S tumors. These tumors spontaneously regress when telomere reserves are exhausted due to the lack of TMM and the inability to counteract telomere shortening pathways (Fig. 3a). Altogether, these findings underscore the critical roles of telomeres and TMM in driving NB malignancy.

Why is HR-NB so strongly connected to telomere maintenance in comparison to other cancers? Studies to date suggest several non-mutually exclusive possibilities. First, in contrast to other cancers, many NB tumors exhibit evidence of telomere trimming [84], a rapid telomere shortening pathway that is utilized by stem cells and germ cells to achieve telomere homeostasis in the setting of high telomerase activity [53, 54]. Second, emerging evidence suggests that HR-NB tumors harbor high levels of DNA damage and exhibit mutation signatures that are consistent with oxidative DNA lesions [23, 84]. In ATRX-deficient HR-NB, an additional mechanism of telomere DNA damage appears to be contributed by replication stress and G-quadruplex formation [85]. Moreover, there is evidence that HR-NB tumors exhibit altered DDR (DNA damage response) [86, 87]. Collectively, the high levels of DNA damage and altered DDR may trigger accelerated DNA loss at telomeres. Taken together, these observations suggest that the need to compensate for the multiplicity of telomere shortening pathways (i.e., incomplete end replication, telomere trimming, and telomere DNA damage) makes it imperative for NB tumors to activate TMMs.

Conversely, if NB tumors fail to activate TMMs and are unable to counter telomere shortening mechanisms, then the tumors are predicted to spontaneously regress. This proposition is consistent with the clinical course of low-risk NB in infants and the lack of TMM in these tumors. Considered in the context of all malignancies, the presence of TMMs in HR-NB is not particularly surprising given that the majority of tumors in most cancer types exhibit either telomerase or ALT [74]. Perhaps more unexpected is the frequent occurrence of low-risk NB that harbors no TMM and that undergoes spontaneous regression. The prevalence of such tumors in NB suggests one of two (non-mutually exclusive) possibilities (Fig. 3b, green box, for low-risk NB): (i) The tumor-initiating cell(s) originally harbor high TMM activities, which become repressed by the time of diagnosis, and (ii) the tumor-initiating cells originally possess very long telomeres and thus have sufficient telomere reserve to undergo numerous cell divisions. Notably, either scenario is compatible with features of neural crest progenitor cells that give rise to NB, which presumably bear characteristics of stem cells, i.e., long telomeres and high telomerase activity [53, 88]. Indeed, the neural crest origin of NB could explain another feature of this cancer, namely the prevalence of “telomere trimming” activity [84]. Although the mechanism of telomere trimming is not well understood, it is known to be particularly active in stem cells and germ cells [53, 54]. Evidence for telomere trimming was also observed in normal neural tissues [84], suggesting that this pathway may remain active in neural development, including in the neural crest progenitors that undergo oncogenic transformation. These considerations underscore the potential impact of NB developmental origin on the telomere biology of this cancer. It is tempting to speculate that the TMM characteristics of tumors in different subgroups of NB may be related to the plasticity road map of the neural crest tumor-initiating cells.

It is worth noting that while TMMs are crucial for the great majority of HR-NB, there are exceptions. In one study, a small subset of HR-NB was found to exhibit no TMM and to manifest continuous telomere erosion while undergoing > 200 population doublings [89]. This is possible because the tumor-derived cells manifested no evidence for telomere trimming and initially harbored extremely long telomeres (Fig. 3b, blue box, for HR-NB). While these exceptions could be interpreted as refuting a universal requirement for TMM in HR-NB, they also provide further illustration of the complex telomere biology of NB progenitor cells and how telomere shortening and lengthening pathways could be dysregulated in different combinations to permit uncontrolled proliferation.

## A reciprocal, mechanistic relationship between telomere regulation and tumor cell differentiation in neuroblastoma

As described in the opening section, another critical determinant of NB malignancy that is dictated by its neural crest origin is tumor cell differentiation. NB displays two major differentiation states that may reflect different stages of neural crest development: undifferentiated MES cells and committed ADRN cells, and these two cell types are interconvertible [14, 17]. Ectopic expression of key transcription factors (e.g., NOTCH and PRRX1), associated with the differentiation program, can trigger the conversion between cell types through a feedforward mechanism [13]. These differentiation states have been implicated in HR-NB malignancy. For example, MES cells are more resistant to the standard NB chemotherapy than ADRN cells in vitro and are enriched after chemotherapy and during relapse in vivo [14, 15, 18, 19].

Even though few studies have addressed the relationship between telomeres and tumor cell differentiation in NB, the fact that both are impacted by its developmental origin suggests a potential mechanistic linkage. Indeed, a recent study uncovered multiple evidence for such a linkage in *MYCN*-amplified HR-NB [17]. First, ADRN and MES cells were found to exhibit sharp differences in telomere-related protein levels and subcellular distributions. Three shelterin components (TRF1, TRF2, and TPP1) and multiple telomere DNA synthesis- or repair-related proteins were consistently detected at higher levels in ADRN cells, and the level of telomerase activity was also higher in these cells [17]. Second, stringent inhibition of telomerase activity by either pharmacologic treatment or the expression of a catalytically inactive TERT (Dn-hTERT) triggered the differentiation of ADRN into MES cell. This differentiation is accompanied by robust changes in the expression of telomere-related proteins that are in line with naturally derived ADRN/MES cells [17]. This again supports telomere remodeling as an integral component of the ADRN/MES switch process. Notably, the conversion induced by telomerase inhibition was reversed upon subsequent up-regulation of telomerase activity. The role of telomerase activity in NB differentiation was also supported by two previous reports. In one study, drug-induced telomerase inhibition resulted in morphologic differentiation as determined by extension of neurites [90]. In the other study, the expression of Dn-hTERT caused NB cells to switch from a neuronal (ADRN-like) to a substrate-adherent (MES-like) morphology with loss of malignant properties [91]. Although these studies did not directly address ADRN-to-MES switch, they provided additional support for a connection between telomerase activity and NB differentiation. Overall, the earlier and recent findings point to a reciprocal mechanistic relationship between tumor cell states and telomere regulation, i.e., ADRN/MES switch engenders telomere/telomerase modulation, and telomerase modulation engenders ADRN/MES switch. The clinical significance of this relationship is currently unclear and worthy of further investigation. In a preliminary exploration, an expression signature that incorporates both telomere- and differentiation-related genes was found to cluster NB patients’ tumors into different groups with distinct clinical outcomes, suggesting potential prognostic applications [17].

## The roles of telomere-related factors in embryonic and neural development: a possible developmental basis for the telomere-differentiation linkage in neuroblastoma

Because normal telomere function is presumably required to stabilize chromosome ends in all cell types, the drastic telomere protein profile differences between ADRN/MES cell types are somewhat unexpected. Could these differences reflect normal changes associated with neural crest development? Indeed, there is growing evidence for both physical and functional alteration of telomere complexes during embryonic and neural development. Moreover, certain telomere proteins (e.g., TRF1 and TRF2) appear to mediate extra-telomeric, differentiation-related functions in normal development. In this section, we will highlight observations concerning telomere proteins in development that could be relevant to their roles in NB tumor cell lineage specification (Table 1). It should be noted, however, that the great majority of these studies address neuronal development and maturation [43, 92, 93] and could not be directly applied to neural crest and NB. They do, however, point to possible connections between telomere proteins and neural crest cell fate that warrant further investigation in the context of NB.

**Table 1:** Differential expression of telomerase and TRF2 in development and differentiation

	ESC	NPC	NGN	MN	Non-neural progenitor cells	References
Telomerase activity						
*Cell culture*						
Human	High activity in embryonic NT2 neuronal precursor cells		Low activity in NT neurons			Kruk et al. [118]
Rat	High activity in NEP stem cells isolated from E10.5 embryos	Low activity in E14.5 neural tubes cells isolated from E14.5 embryos (mixed cell of postmitotic neurons, neuronal precursors, NEP cells, and glial precursors)			High telomerase activity in glial precursor cells	Cai et al., 2002*
*Embryo*						
Mouse	High activity at E13 (embryonic day 13) during brain development progressive decline from E13 to E18 low level until P3 (postnatal day 3) undetectable telomerase activity from P16					Klapper et al. [117]
TRF2						
*Cell culture*						
Human	Low levels	High levels			Low in FC and HPC	Ovando-Roche et al. [43]
Mouse		Undetectable	Undetectable	High		Cheng et al. [97]
	Low or Undetectable	Low or Undetectable				Ovando-Roche et al. [43]
Rat		Detectable	Undetectable in differentiated neural cells Undetectable in cortical neuron		High in glial cells	Zhang et al. [41]
*Embryo*						
Mouse	Undetectable in NPCs and NGNs at E16 during brain development Very low in neurons at E18 Progressively higher levels through P15 High levels in adult brain					Cheng et al. [97]
Rat	High at E14 during brain development Very low after E14 through adult brain					Zhang et al. [41]

*ESC* embryonic stem cell, *NPC* neural progenitor cell, *NGN* newly generated neuron, *MN* mature neuron, *NT2* teratocarcinoma cells with CNS neuronal precursor features, *NT* postmitotic CNS neurons, *NEP* neuroepithelial cells, *FC* human fibroblast, *HPC* hepatocyte progenitor cell

*Cai J., Wu Y., Mirua T., Pierce J. L., Lucero M. T., Albertine K. H., et al. (2002). Properties of a fetal multipotent neural stem cell (NEP cell). Dev. Biol. 251, 221–240

### TRF2

The telomere protein with the strongest evidence for development- and neural differentiation-related changes is TRF2, a key component of the shelterin complex [46, 94]. In non-neuronal cells, TRF2 can prevent senescence and apoptosis by protecting telomeres and inhibiting telomere-associated DNA damage response involving ATM and p53 [94–96]. The special involvement of TRF2 in the nervous system was suggested by regulated expression of TRF2 during neural development and differentiation in animals and cell cultures (Table 1). In general, TRF2 expression was found to be up-regulated during neuronal differentiation and subsequently maintained throughout neuronal maturation [97]. In mouse and human stem cell or progenitor cell culture models, TRF2 was initially expressed at low levels and underwent strong increase following differentiation into neural progenitor cells or neurons [41, 43, 97].

The mechanistic connections between TRF2 and neural development were further reinforced by functional studies in stem and progenitor cells. For example, shRNA-mediated knockdown of TRF2 in hESCs (human embryonic stem cells) and NPCs (neural progenitor cells) had strong inhibitory effects on neural differentiation [43]. Interestingly, these inhibitory effects were not associated with telomere length changes. Instead, current evidence suggests that TRF2 promotes neural differentiation by modulating the function of REST (repressor element-1-silencing transcription factor), a repressor of neuronal gene expression [98, 99]. This TRF2-REST pathway is multi-faceted, context-dependent, and involves the activities of not only full-length proteins, but also short isoforms of both proteins. In several studies, TRF2 was observed to promote neural differentiation by binding to and up-regulating REST4, a truncated isoform that promotes rather than inhibits neural gene expression [43, 100]. Consistent with this proposal, overexpressing TRF2 increased hREST4 levels in hESCs, whereas knockdown of TRF2 reduced hREST4 in NPCs. Notably, the failure of shTRF2-treated NPCs to differentiate was rescued through hREST4 overexpression, suggesting that TRF2 may act upstream of and primarily through hREST4 [43]. Another mechanism by which TRF2 may promote neural differentiation involves a truncated, neural tissue-specific isoform named TRF2-S. This splicing variant, which contains a unique nuclear export signal, was found to be predominantly cytoplasmic and to be complexed with full-length REST [41]. TRF2-S was thus proposed to enhance neuronal maturation by sequestering REST in the cytoplasm and preventing the latter’s ability to suppress neural gene expression. In support of this idea, co-expression of TRF2-S (but not full-length TRF2) with REST blocked the neural gene-silencing effect of REST in a cell culture model [41]. Besides these mechanisms, TRF2 is also reported to bind non-telomeric regions of the genome and regulate the transcription of associated genes [101–103]. Moreover, a recent report suggests that the neural developmental functions of TRF2 is conserved in zebrafish [104].

The functions of TRF2 in the development of the nervous system suggest a role for this protein in NB differentiation and cell fate specification. In line with this idea, TRF2-S was found in both ADRN and MES cells, consistent with the neural crest origin of this tumor [17]. In addition, ADRN cells evidently contain higher levels of TRF2 that are predominantly cytoplasmic, again consistent with the neuroblast-like phenotype of these cells. Conversely, the low level of TRF2 in MES cells suggests that these cells may resemble ESC with regard to telomere regulation (Table 1). Quite strikingly, two recent studies of mouse ES cells point to dramatically different function of TRF2 in these cells; in contrast to somatic cells, TRF2 deletion in ES cells triggers only a mild DDR and no telomere fusions [105, 106]. This reduced telomere protection function of TRF2 appears to be due to increased role of other telomere proteins (e.g., POT1B and ZSCAN4) or functional redundancy. It is possible that the neural crest-derived mesenchymal cells may share this reduced dependency on TRF2 and thus manifest reduced expression. Accordingly, neuroblastoma MES cells may resemble normal neural crest mesenchymal cells with regard to telomere regulation.

### TRF1 and TERF2IP

TRF1 is the second major duplex telomere-binding protein in shelterin. It probably arose through duplication of an ancestral TRF2-like gene during vertebrate evolution [107]. While the evidence linking TRF1 to neural development is not as strong as that for TRF2, there are multiple studies that support a potential connection. TRF1 is strongly implicated in stem cell function and pluripotency; its expression is up-regulated in ESCs and iPSCs, but may be down-regulated in neural progenitor cells in vitro [43, 108, 109]. Knocking down TRF1 reduced the reprogramming potential of stem cells in vivo, further underscoring its roles in maintaining pluripotency. The stem cell function of TRF1 could be mediated through its established roles in promoting telomere replication and genome stability [110, 111]. Alternatively, like TRF2, TRF1 has been suggested to bind interstitial regions of the chromosome and could mediate its functions through a non-telomeric pathway [102]. However, a genome-wide analysis of TRF1-binding to chromatin in mouse embryonic fibroblasts points to exclusive localization of TRF1 to telomeric regions [112]. Like TRF1, TERF2IP has been reported to mediate several non-telomeric functions such as metabolism by regulating transcription [113, 114]. Several recent studies further implicate TERF2IP in hematopoietic stem cell survival, but no direct connection to neural development has come to light [115, 116].

### Telomerase and TERT

Telomerase activity is present at high levels in neural progenitor cells and newly generated neurons, but declines rapidly after terminal cell division, suggesting a role in neural development [117, 118] (Table 1). The association between telomerase activity and neural cell differentiation is supported by several cell culture studies. For example, differentiation of neural cell lines and primary neurons correlates with a decrease in telomerase activity [44, 119]. Conversely, anti-sense inhibition of telomerase activity in glioma cells can promote the expression of glial cell markers in surviving cells [120]. Abnormally short telomeres in neural stem cells have been shown to disrupt neuronal differentiation [121]. However, studies of *mTERT*-/- mice have led to conflicting conclusions regarding the need for TERT in neurodevelopment [121–123]. The potential functions of telomerase and TERT in neural crest development and how these functions relate to the linkage between telomerase and NB tumor cell differentiation warrant further investigation.

## Implications for future research and for developing telomere-based biomarkers and therapies for HR-NB

In short, the critical importance of telomeres in NB malignancy is underscored by (i) the poor prognosis of tumors that harbor telomerase or ALT and (ii) the spontaneous regression and/or maturation of low stage tumors that harbor no TMM. It should come as no surprise that TMMs have emerged as a high priority focus for HR-NB therapeutic development [87, 124]. However, as noted in the 2020 Trans-Atlantic Neuroblastoma NDDS (New Drug Development Strategy) initiative, there are currently no drugs specifically related to telomere maintenance (e.g., drugs that target telomerase, ALT, TERT, or ATRX) that are being tested clinically [124]. One obstacle for telomerase-based therapies is toxicity in normal tissues and cells that harbor this enzyme [125]. Nevertheless, promising preclinical data for compounds that target telomeres are beginning to emerge, and further development of these compounds is clearly worthwhile [87, 126, 127]. At the same time, since the role of telomeres in HR-NB is multi-faceted, additional preclinical studies that address the telomere-related vulnerabilities of NB as well as the connections between telomeres and other determinants of NB malignancy should help prioritize these early leads.

One area that merits better understanding is the roles played by ADRN/MES interconversion and telomeres in the context of standard chemotherapy and radiotherapy. It is possible, for example, that the frequent response of HR-NB patients to front-line therapies may be due in part to trans-differentiation of faster growing ADRN into slower growing MES cells. The MES cells could become dormant, only to regrow with the cessation of chemotherapy, a pattern not uncommon among patients after favorable initial responses. The prevalence of relapse and progression in HR-NB suggests that the extent of ADRN/MES conversion and related TMM alterations (e.g., reduction in telomerase activity) is not sufficient to eradicate tumor growth. This idea is also consistent with the conclusion, based on analysis of 20 paired tumor samples, that the TMM status does not change significantly over the course of disease [21]. Thus, the inability of conventional therapies to improve survival may be partly attributable to their failure to fully suppress TMM.

Another set of factors to consider in relation to NB telomeres are the levels of DNA damage and the proficiency for DNA repair. The impact of DNA damage in the initiation and evolution of NB is evidenced by the discovery of mutational signatures associated with specific DNA damage pathways [23]. Among these signatures, the ROS (reactive oxygen species)-associated SBS18 appears to be especially prevalent [128, 129]. Consistent with this finding, glycosylase-sensitive lesions that inhibit PCR amplification of telomere DNA have been detected in HR-NB [84]. Notably, oxidative stress has been repeatedly implicated in generating telomere DNA damage and triggering accelerated telomere shortening [55]. The high levels of ROS-induced telomere lesions in NB thus provide an additional reason for the heightened dependence of these tumors on TMMs. Moreover, if ROS indeed plays a role in the addiction of NB tumors to TMMs, it may be another surrogate biomarker, together with the SBS18 signature, for telomere-directed therapies. Not to be overlooked is the complex relationship between DNA repair activities and telomere maintenance. While oxidative lesions such as 8-oxoguanine can be eliminated by BER (base excision repair) pathways, incomplete repair of such lesions could lead to accumulation of intermediates (e.g., single strand breaks) that are more toxic to the cell. Therefore, therapeutic strategies that target DNA damage response and DNA repair factors should be evaluated also in connection with their impacts on telomeres.

Finally, the effects of targeting telomeres on the immunobiology of NB are worth considering. To date, the only clinically validated immunotherapies for NB are monoclonal antibodies against GD2, a disialoganglioside that is enriched on neuroblasts. While other approaches such as immune checkpoint inhibitors and CAR-T cell therapy showed promise, their efficacy in neuroblastoma needs to be confirmed in larger phase III trials [130–134]. Notably, two recent reports have revealed striking differences in the immunogenicity of ADRN and MES cells that could have strong implications for immunotherapeutic strategies [135, 136]. Particularly noteworthy is the greater immunogenicity manifested by the MES cell type, including both innate and adaptive immune gene signatures as well as evidence for inflammatory sensing. It will therefore be of great interests to determine the effect of telomere-targeting drugs on the immunogenicity of tumor cells. Understanding the interconnection and consequences of telomere-based therapies on NB cell proliferation, differentiation, DNA damage/repair, and immunogenicity is critical not only for eventually bringing these therapies to the clinic, but also for developing more effective ways to combine them with standard of care and other investigational modalities.

The issues and concepts we have discussed for neuroblastoma may be applicable to other pediatric cancers. In contrast to adult cancers, pediatric cancers have a different mutational landscape and are strongly influenced by the developmental origin and stage of the cancer-initiating cells [137]. Given the precedence established by NB, one may speculate that telomere-related factors could likewise influence the differentiation states of other pediatric cancers such as medulloblastoma and sarcomas, which also display TMM activation and cellular plasticity [138–141]. Telomere-targeting therapies for NB and the research tools already developed to study telomeres in NB could provide the paradigm and the toolbox for undertaking similar initiatives in other pediatric cancers.

## Data Availability

Not applicable because no data or material is generated for this work.
